# Novel antimicrobial 3-phenyl-4-phenoxypyrazole derivatives target cell wall lipid intermediates with low mammalian cytotoxicity

**DOI:** 10.1038/s41598-025-19561-y

**Published:** 2025-10-13

**Authors:** Blanca Fernandez-Ciruelos, Marco Albanese, Femke Taverne, Paul W. Finn, Jerry M. Wells

**Affiliations:** 1 Host-Microbe Interactomics Group, Wageningen University & Research (WUR), de Elst 1, 6708 WD Wageningen, The Netherlands; 2 Oxford Drug Design (ODD), Oxford Centre for Innovation, Blue Boar Court, 9 Alfred St, Oxford, OX1 4EH UK; 3 School of Computer Science, University of Buckingham, Hunter Street, Buckingham, MK18 1EG UK

**Keywords:** Pathogens, Antimicrobials, Antibiotics, Antimicrobial resistance, Drug discovery

## Abstract

The growing crisis of antimicrobial resistance (AMR) underscores the critical need for innovative antimicrobial discoveries. Novel antibiotics targeting the bacterial cell wall remain an attractive area of research, due to their conservation and essentiality in bacteria and their absence in eukaryotic cells. Antibiotics targeting lipid II are of special interest due to the reduced potential for target modification of lipid components and their surface accessibility to inhibitors. In this study, we identified 3-phenyl-4-phenoxypyrazole analogues named PYO12 and PYO12a with bactericidal activity against gram-positive bacteria and low cytotoxicity for different types of mammalian cells. Gram-negative bacteria were resistant to PYO12 activity through extrusion of this compound via efflux pumps. Exposure to PYO12 induces expression of genes involved in resistance to antimicrobials targeting the cell wall, suggesting that PYO12 acts via binding to lipid II or other lipid intermediates involved in peptidoglycan or teichoic acid biosynthesis. Antagonism of PYO12 antibacterial activity by undecaprenyl-pyrophosphate supports the idea that PYO12 may bind to the lipid moiety of lipid II blocking the shuttling of peptidoglycan precursors across the cytoplasmic membrane. These findings open opportunities to further develop these compounds as antibiotics targeting bacterial cell wall synthesis.

**Supplementary Information:**

The online version contains supplementary material available at 10.1038/s41598-025-19561-y.

## Introduction

Antimicrobial resistance (AMR) has recently become one of the leading causes of death worldwide^1^ highlighting the need for new antibiotics with low potential for resistance development. Bacterial cell wall synthesis is still one of the most desirable targets for antibiotic development due to its essentiality for bacterial growth and the lack of similar structures in mammalian cells. Antibiotics targeting the bacterial cell wall mainly act through inhibition of cell wall synthesis enzymes (e.g. ampicillin) or binding to cell wall building blocks such as lipid II (e.g. vancomycin or nisin)^2^.

Lipid II is an essential precursor in peptidoglycan biosynthesis in all bacteria. It is initially synthesized on the inner leaflet of the cytoplasmic membrane, where its undecaprenyl-pyrophosphate (C_55_-PP) hydrocarbon chain acts as a lipid anchor. During peptidoglycan synthesis, lipid II shuttles across the membrane to incorporate its disaccharide MurNAc-GlNAc-pentapeptide ʻbuilding blockʼ into the growing peptidoglycan polymer. The lipid intermediate C_55_-PP is then dephosphorylated and translocated back to the cytosolic side by a yet unknown mechanism where the formation of lipid II starts again^3^. Lipid II formation is a limiting step in cell wall synthesis due to the fixed number of undecaprenyl-phosphate (C_55_-P) molecules per cell (2 × 10^5^ molecules)^4,5^. The essentiality of lipid II, its high conservation in bacteria and its accessibility to inhibitors on the outside of the cytoplasm makes it a highly attractive target for antibiotic development. One of the main disadvantages of antibiotics targeting lipid II is that they are usually not effective against Gram-negative bacteria, mainly due to the low permeability of the LPS populated outer membrane and the presence of multiple efflux pumps^6^.

The main lipid II binders used in the clinic are glycopeptides, the most well-known being vancomycin that binds to lipid II through the carboxyl end of the pentapeptide D-Ala-D-Ala^7^ however resistance has risen due to the substitution of D-Ala-D-Ala by D-Ala-D-Lactate^8^. Other lipid II binding antibiotics, such, nisin, and cyclic depsipeptides^9^ recognize the C_55_-PP moiety of the lipid II, while other antibiotics such as bacitracin^10^ directly bind to the lipid intermediate C_55_-PP preventing lipid II formation. An advantage of targeting C_55_-PP is that this molecule is also used for teichoic acid^11^ and capsule biosynthesis^12^ so various pathways can be inhibited simultaneously. Resistance to these antibiotics has been reported by the induction of ABC transporters VraDEH and BraDE, which play a role in antibiotic transport and detoxification^13^ but resistance due to modification of the C_55_-PP lipid carrier has not been reported presumably due to the difficulty for bacteria to sufficiently modify the lipid moiety and retain functionality^14^.

The discovery of small molecules able to bind to the lipid moiety of the lipid II to surpass resistance emergence in Gram-positive bacteria has drawn a lot of interest in recent years. Different small molecules have been found to bind to the undecaprenyl moiety of lipid II^15–18^ however, cytotoxicity has remained an issue for these molecules (with selectivity indexes, i.e., ratio between the compound’s antibacterial activity and its cytotoxicity, between 1.2^16^ and 4^17^ for small molecules targeting the lipid moiety of lipid II).

Our previous work showed 3,4-diphenylpyrazoles (DPP) to be candidate antimicrobials with a mechanism of action related to or caused by loss of inner membrane integrity in a similar manner to nisin^19^. Here, we further explored the DPP scaffold and found a 3-phenyl-4-phenoxypyrazole (PYO) derivative referred to as compound **PYO12** and the related **PYO12a**, with low cytotoxicity in vitro and bactericidal activity against a panel of Gram-positive bacteria. We demonstrated that exposure to **PYO12** leads to loss of bacterial membrane integrity and hypothesize that it acts in a similar manner to nisin by binding to lipid II or other lipid intermediates. To corroborate the mechanism of action, we quantified the expression of cell wall stress genes *vraX* and *cwrA* which are known to be significantly upregulated by agents that target the bacterial cell wall or membrane, such as bacitracin, vancomycin and antimicrobial peptides (AMPs)^18,20,21^. We also analysed the expression of the regulons controlled by two-component systems (GraRS, BraRS, and VraRS), which have been linked to decreased susceptibility to antibiotics targeting lipid II and the bacterial cell wall^22^. **PYO12** induced expression of cell wall stress genes and our studies showing antagonism of antibacterial activity by C_55_-PP indicate that **PYO12** binds to the C_55_-PP moiety of lipid II and is a promising lead for the development of a novel antibiotic.

## Results

### Antimicrobial activity of PYO derivatives against *S. aureus*

Starting with the DPP scaffold previously published^19^ we identified a set of commercially available analogues (**PYO1-PYO22**, Table 1) with a 4-phenoxy group instead of the original 4-phenyl group. We then assessed their antibacterial activity against *S. aureus* str. Newman. The most potent derivatives reached a minimal inhibitory concentration (MIC) of 1 µg/ml (**PYO1** and **PYO12**, Table 1). In the DPP series, replacing the R_1_ methyl group with a trifluoromethyl group improved antibacterial activity. To explore if the DPP’s SAR could be transferred to the novel series, we synthesized **PYO23** (Figure SF2), the trifluoromethyl analogue of one of the most potent compounds of the series **PYO1**. In contrast to the original series, this transformation resulted in a decrease in antibacterial potency.

The activity of **PYO14** suggests that the replacement of the R_2_-hydroxyl of ring A, a resorcinol, with a methoxy group is tolerated. The improved activity of the R_4_-propyloxy derivative **PYO20** and the R_4_-benzyloxy **PYO11** compared to their hydroxy analogues (**PYO18** and **PYO13**, respectively) indicate that O-alkylation at this position can be beneficial in terms of antibacterial activity. The matched molecular pair **PYO1/PYO4** shows that the R_5_-ethyl substitution causes a marked improvement in activity. Methylation is tolerated at the R_3_-position (**PYO19**), whilst the R_6_-methyl derivative is only weakly active (**PYO22**).

Favourable substitutions on the C-ring are a chlorine or isopropyl in position R_7_ (**PYO1**, **PYO17**, **PYO18**, **PYO20**, **PYO23**/**PYO2**), a phenyl in position R_8_ (**PYO12**) and a bromine in position R_9_ (**PYO14**). We also investigated the impact of exchanging the -O-linker with a -S-linker by synthesizing **PYO24** (Figure SF3). Its antimicrobial potency was similar to the parent analogue **PYO1**, indicating that the replacement is bioisosteric.

**Table 1:** Chemical structure, antibacterial activity against *S. aureus* and cytotoxicity in HEK293 cells of a series of 3-phynyl-4-henoxypyrazoles and common antimicrobials (nisin, vancomycin and novobiocin). The MIC against S. aureus and LC_50_ (concentration causing 50% of cell death in HEK293 cells) are indicated in micrograms per milliliter. Compound (Comp.) substitutions (R1 to R9) on rings A, B or C of the scaffold, as indicated in the diagram, are listed. Selectivity index (S.I.) is calculated as MIC/LC_50_.*-O-linker substituted by -S-linker.


Compound name	*R*_1_	*R*_2_	*R*_3_	*R*_4_	*R*_5_	*R*_6_	*R*_7_	*R*_8_	*R*_9_	MIC S. aureus (µg/ml)	LC_50_ HEK293 (µg/ml)	S.I.
PYO1	-Me	-OH	-H	-OH	-Et	-H	-Cl	-H	-H	1	11.23	11.23
PYO2	-Me	-OH	-H	-OH	-H	-H	-iPr	-H	-H	8	14.78	1.84
PYO3	-CF_3_	-OH	-H	-OH	-H	-H	-H	-H	-H	16	22.36	1.397
PYO4	-Me	-OH	-H	-OH	-H	-H	-Cl	-H	-H	32	43.6	1.36
PYO5	-Me	-OH	-H	-OH	-H	-H	-OEt	-H	-H	64	26.75	0.411
PYO6	-H	-OH	-H	-OMe	-H	-H	-COOH	-H	-H	> 128	> 128	N.A.
PYO7	-Me	-OH	-H	-OH	-H	-H	-COOMe	-H	-H	> 128	19.73	N.A.
PYO8	-Me	-OH	-H	-OH	-H	-H	-OMe	-H	-H	64	17.36	0.271
PYO9	-H	-OH	-H	-OMe	-H	-H	-H	-H	-OEt	32	23.59	0.737
PYO10	-H	-OH	-H	-OBn	-H	-H	-OMe	-H	-H	64	8.46	0.132
PYO11	-H	-OH	-H	-OBn	-H	-H	-H	-OMe	-H	16	10.69	0.668
**PYO12**	**-Me**	**-OH**	**-H**	**-OH**	**-H**	**-H**	**-H**	**-Ph**	**-H**	**1**	**15.05**	**15.05**
PYO13	-H	-OH	-H	-OH	-H	-H	-H	-OMe	-H	> 128	32.3	N.A.
PYO14	-H	-OMe	-H	-OH	-H	-H	-H	-H	-Br	4	7.4	1.85
PYO15	-H	-OH	-H	-OH	-H	-H	-F	-H	-H	> 128	> 128	N.A.
PYO16	-Me	-OH	-H	-OEt	-H	-H	-H	-H	-H	> 128	46.7	N.A.
PYO17	-H	-OH	-H	-OMe	-H	-H	-Cl	-H	-H	4	28.9	7.22
PYO18	-H	-OH	-H	-OH	-H	-H	-Cl	-H	-H	8	16.4	2.05
PYO19	-Me	-OH	-Me	-OEt	-H	-H	-F	-H	-H	16	11.9	0.74
PYO20	-H	-OH	-H	-OPr	-H	-H	-Cl	-H	-H	2	9.4	4.7
PYO21	-H	-OH	-H	-OMe	-H	-H	-H	-H	-F	32	7.3	0.228
PYO22	-Me	-OH	-H	-OMe	-H	-Me	-H	-H	-H	128	24.9	0.195
PYO23	-CF_3_	-OH	-H	-OH	-Et	-H	-Cl	-H	-H	4	28.5	7.12
PYO24*	-Me	-OH	-H	-OH	-Et	-H	-Cl	-H	-H	2	25.64	12.82
Nisin										6.25	210.43	33.66
Vancomycin										0.5	220.5	441
Novobiocin										0.5	115.20	230.4

### Cytotoxicity of antimicrobial PYOs

The cytotoxicity of the PYOs (Table 1) was tested using the human kidney cell line HEK293 and calculated as LC_50_ (lethal concentration 50, i.e. the concentration required to kill 50% of the cell population). All the compounds with antibacterial activity against *S. aureus* str. Newman were toxic to mammalian cell lines at low concentrations with only three compounds (**PYO1**, **PYO12** and **PYO24)** showing a selectivity index (drug MIC/LC_50_) higher than 10. We decided to further investigate the SAR of **PYO12** and its antimicrobial mechanism because it had a different structure to other compounds of the series due to the presence of a phenyl group at position R_8_. The cytotoxicity of **PYO12** for the liver cell line HepG2, commonly used in toxicology studies, gave LC_50_ value of 11.5 ± 1.33 µg/ml, and selectivity index of 11.5. To allow the comparison between PYO derivatives and commonly used antimicrobials, the toxicity of nisin, vancomycin and novobiocin was also calculated, as well as their selectivity indexes.

Finally, we tested the potential damaging effects of **PYO12** on biological membranes using the sensitive red blood cell haemolysis assay. Incubation of 150 µg/ml of **PYO12** for 30 min with sheep red blood cells in PBS resulted in complete haemolysis. However, haemolysis dropped below 10% at 75 µg/ml and to 0% at 37.5 µg/ml. The HC_50_ (concentration that causes 50% haemolysis in RBCs) was calculated to be 121 µg/ml, resulting in a selectivity index of 60.5 (Supplementary Figure SF1). The three mentioned antimicrobials did not show haemolysis in the highest concentrations tested of 250 µg/ml.

Since PYO structures are derived from known Hsp90 inhibitors (mammalian chaperone) we tested the inhibition of Hsp90 by **PYO12**, however no inhibition was detected. This was expected due to the bulky substitution of **PYO12** probably leading to reduced affinity to the ATP domain^19^.

### Spectrum of activity

The selectivity index of compound **PYO12** prompted us to test its antimicrobial activity against *Bacillus subtilis* and a panel of common opportunistic human pathogens, methicillin-resistant *Staphylococcus aureus* (MRSA), vancomycin susceptible and resistant *Enterococcus faecium* and *Enterococcus faecalis*, *Streptococcus pneumoniae*,* Escherichia coli* and *Pseudomonas aeruginosa*, and *Streptococcus suis* a common pig pathogen (Table 2). Compound **PYO12** showed inhibitory activity against all Gram-positive bacteria tested with MICs ranging from 1 to 16 µg/ml with *E. faecalis* being the least susceptible Gram-positive species. **PYO12** had no inhibitory effect against Gram-negative *E. coli* and *P. aeruginosa.*

**Table 2:** Minimal inhibitory concentration of **PYO12** for different species and strains.

Bacterial species	MIC (µg/ml)
*S. aureus*	1
*S. aureus* (MRSA)	4
*E. faecium*	8
*E. faecium vanA* ^*+*^	4
*E. faecium vanB* ^*+*^	4
*E. faecium vanAvanB* ^*+*^	4
*E. faecalis*	16
*E. faecalis vanA* ^*+*^	8
*B. subtilis*	1
*S. suis*	4
*S. pneumoniae*	1
*E. coli*	> 128
*P. aeruginosa*	> 128

### The TolC-dependent efflux pumps are mainly responsible for the lack of efficacy of PYO12 in Gram-negative bacteria

We hypothesized that the lack of activity against Gram-negative bacteria was due to reduced permeability caused by the outer membrane and/or effective efflux of the compounds. We investigated the antibacterial activity of the compounds against *E. coli* (D21f2), a strain with truncated a lipopolysaccharide (LPS) barrier, and a *tolC* mutant of *E. coli* (JW5503), a strain defective in compound efflux.

Whilst none of the compounds inhibited WT *E. coli* ATCC25922 growth, some derivatives showed improved activity against *E. coli* D21f2, with MIC as low as 16 µg/ml for **PYO17** and **PYO18**. However, the MIC remains high (32–128 µg/ml) for most compounds (Table 3), indicating that the LPS layer has a limited impact on compound penetrability. The MIC for *E. coli* Δ*tolC* was lower or the same as for *S. aureus* for 12 of 21 active compounds (Table 3) indicating a key role for efflux pumps in the efflux of **PYO12** and related compounds in *E. coli.* In the cases where the MIC was significantly higher for *E. coli* Δ*tolC* than for *S. aureus*, an ethyl or isopropyl group substitution was present, suggesting these groups reduce penetrability through the outer membrane (compounds **PYO1**, **PYO2**, **PYO19**, **PYO20**, Table 1).

**Table 3:** Minimal inhibitory concentration of PYO derivatives against *E. coli* outer membrane and efflux mutants. The MIC for Wild-type (WT) *E. coli* ATCC 25,922, *E. coli* D21f2 with a truncated LPS layer and *E. coli* Δ*tolC* (JW5503) lacking TolC a crucial protein component of efflux pumps, and the Gram-positive *S. aureus.*

Compound	MIC S. aureus (µg/ml)	MIC E. coli ΔtolC (µg/ml)	MIC E. coli D21f2 (µg/ml)	MIC E. coli WT (µg/ml)
PYO1	1	32	> 128	> 128
PYO2	8	32	64	> 128
PYO3	16	8	32	> 128
PYO4	32	16	128	> 128
PYO5	64	32	> 128	> 128
PYO6	> 128	128	> 128	> 128
PYO7	> 128	> 128	> 128	> 128
PYO8	64	64	128	> 128
PYO9	32	16	> 128	> 128
PYO10	64	128	> 128	> 128
PYO11	16	64	> 128	> 128
PYO12	1	2	> 128	> 128
PYO13	> 128	32	64	> 128
PYO14	4	16	128	> 128
PYO15	> 128	16	64	> 128
PYO16	> 128	64	> 128	> 128
PYO17	4	2	16	> 128
PYO18	8	2	16	> 128
PYO19	16	> 128	> 128	> 128
PYO20	2	16	64	> 128
PYO21	32	8	32	> 128
PYO22	128	> 128	> 128	> 128
PYO23	4	16	64	> 128
PYO24	2	8	64	> 128

### Structure-activity relationship of PYO12 derivatives

**Fig. 1:**
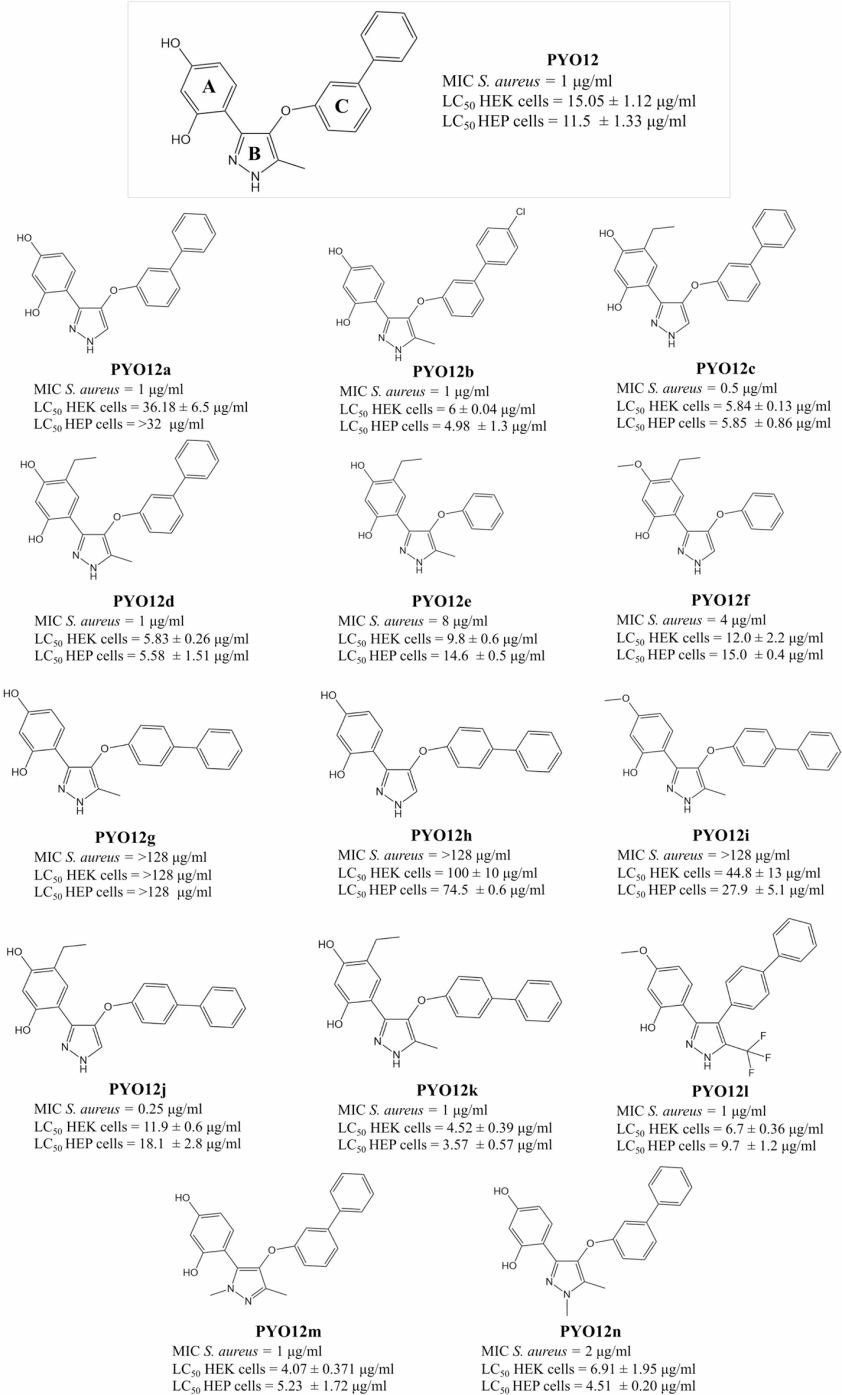
Structure of fourteen **PYO12** analogues found using similarity search. A, B and C rings are indicated in **PYO12** structure. Minimal inhibitory concentration (μg/ml) against *S. aureus* and LC50 (μg/ml) for HEK293 and HepG2 cells is included.

To further study the SAR of **PYO12**, we purchased analogues from commercial vendors (**PYO12e-f**, **PYO12i** and **PYO12l**, Molport, Riga) or performed targeted synthesis (**PYO12a-d**,** PYO12g-h**, **PYO12j-k**, **PYO121m-n**, O2H group, Oxford, supplementary data, Figure SF4-SF13). With respect to the SAR (Fig. 1), elimination of the R_1_-methyl group (general scaffold in Table 1) in the pyrazole ring (B ring) improves antibacterial activity when combined with the R_5_-ethyl group (**PYO12c/PYO12d** and **PYO12j**/**PYO12k**), whilst the MIC in unchanged in the R_5_-unsubstituted matched molecular pair **PYO12a**/**PYO12**. In all three cases removing the R_1_-methyl increases the selectivity index over cytotoxicity in the two listed cell lines. N-methylation of the pyrazole increases cytotoxicity, but it does not alter the MIC, indicating that the hydrogen bond donor in the ring B is not essential for the antibacterial activity (compare **PYO12** to **PYO12n** and **PYO12m**).

A distinctive feature of **PYO12** is the presence of a phenyl in position R_8_. The addition of a chlorine to the para position of this phenyl does not have a significant effect on the activity (compare **PYO12** vs. **PYO12b**). Removal of the R_8_-phenyl is detrimental for the antimicrobial activity (see pair **PYO12d**/**PYO12e**). Moving the phenyl to the R_7_ position results in a loss of antibacterial activity (see pairs **PYO12**/**PYO12g** and **PYO12a**/**PYO12h**). However, potency is recovered when substituting the R_5_ position with an ethyl (**PYO12c**/**PYO12j** and **PYO12d**/**PYO12k**). The potency of **PYO12l** suggests that the O-spacer is not required for activity.

In this set of compounds, **PYO12j** stands out for the excellent MIC 0.25 µg/ml. **PYO12j** cytotoxicity in HepG2 and HEK cells is 11.9 ± 0.6 µg/ml and 18.1 ± 2.8 µg/ml, respectively, giving selectivity indexes of 47.6 and 72.4, which are reasonable for optimization. **PYO12a** is the derivative with best profile, as it maintains the antibacterial potency of **PYO12** and shows low cytotoxicity in both cell lines.

### Compound PYO12 is bactericidal and increases bacterial membrane permeability

**Fig. 2:**
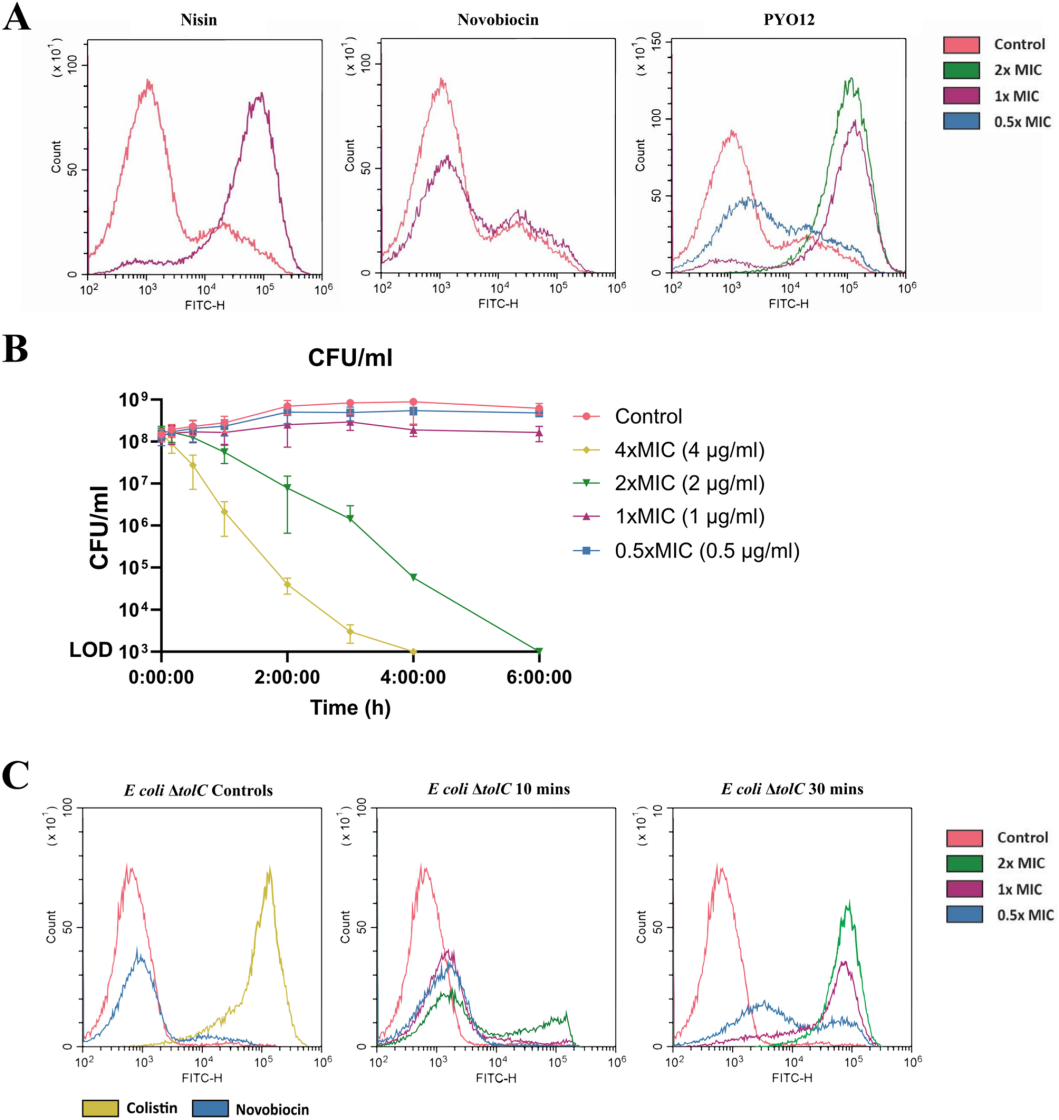
Membrane permeability and time-kill curve in *S. aureus* and *E. coli* ΔtolC by **PYO12**. (**A**) Membrane integrity assessed by SYTOX Green uptake and analysed by flow cytometry. Overlay histograms represent fluorescence intensity of stained bacterial cells (event count vs FITC fluorescence). Increased FITC fluorescence (488 nm) of bacterial population occurs after 5 min exposure to 0.5, 1 and 2xMIC of **PYO12** compared to the non-treated control and negative control treated with novobiocin (1xMIC). Similar shifts in fluorescence are observed with the positive control nisin (1xMIC), a known membrane permeabilizing antimicrobial. (**B**) Time-kill curve of *S. aureus* incubated with PYO12 at 0.5, 1, 2 and 4xMIC (0.5-4 μg/ml). Graphs show CFU/ml vs time after addition of **PYO12**. Error bars show standard deviation (SD) of three independent experiments. (**C**) Membrane integrity after 10 min and 30 min exposure to 0.5, 1 and 2xMIC of **PYO12** to *E. coli* ΔtolC compared to the non-treated control. Negative control (novobiocin, blue) and positive control (colistin, yellow) are included.

Previous studies showed that pyrazole derivatives compromise *S. aureus* membrane integrity^19^. To investigate if this is the putative mechanism of action of compound **PYO12** we performed assays using SYTOX Green, a dye which only enters the bacteria and stains nucleic acids when membrane permeability is compromised. Like nisin which was used as a positive control, addition of 1xMIC (1 µg/ml) of **PYO12** to *S. aureus* for 5 min increased membrane permeability (Fig. 2A).

To rule out the possibility that we were measuring dead bacteria after exposure to **PYO12**, we performed a time-kill curve assay with different concentrations of **PYO12** (Fig. 2B). At 1xMIC (1 µg/ml), **PYO12** has a bacteriostatic effect (Fig. 2B). At 2xMIC (2 µg/ml) **PYO12** is bactericidal after 30 min exposure, and this effect is more pronounced with 4xMIC. From these results we concluded that at 1xMIC, **PYO12** increases membrane permeability prior to any loss of viability.

Finally, to show that the mechanism of action in Gram-negative bacteria is also related to disruption of the inner membrane, we performed the membrane damage assay using *E. coli* Δ*tolC* (Fig. 2C). Even though the Gram-negative *E. coli* Δ*tolC* strain needs a higher exposure time to show membrane damage, we can see that after 30 min, 1xMIC exposure to **PYO12** (2 µg/ml), showed similar membrane damage as the colistin control (1xMIC = 4 µg/ml).

### Putative mechanism of action of PYO12 involves binding to lipid II or other cell-wall lipid intermediates

**Fig. 3:**
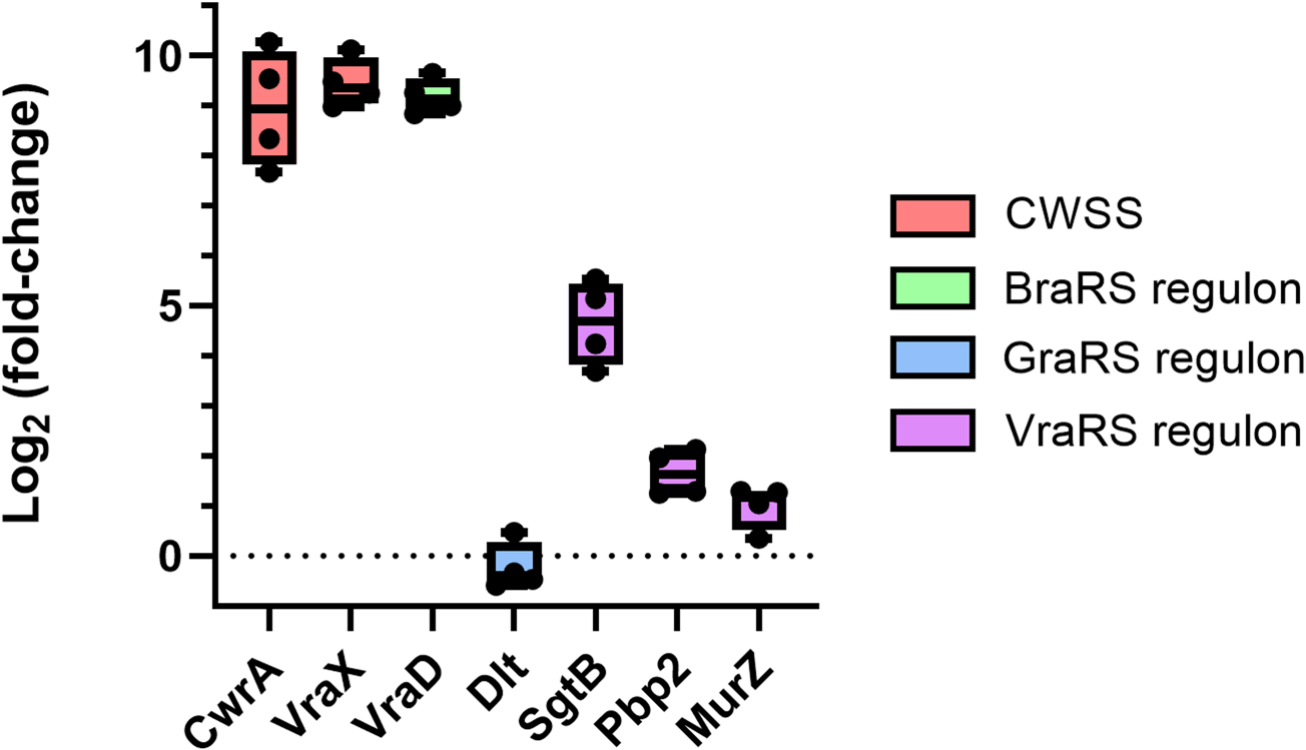
Differential expression of genes involved in response to cell-wall targeting antibiotics in *S. aureus* exposed to 1 μg/ml of **PYO12**. In red *cwrA* and *vraX* are genes generally involved in the cell wall stress stimulon (CWSS), in green *vraD* is regulated by the TCS BraRS, in blue dltA regulated by the TCS GraRS, and in violet *sgtB*, *pbp2* and *murZ* which are regulated by the TCS VraRS. Differential expression is expressed as log_2_ of the fold change as calculated by qPCR and the 2^−ΔΔCt^ method.

To provide further evidence that **PYO12** works by targeting cell-wall or bacterial membrane, expression of genes involved in cell wall stress was quantified by PCR. Exposure of *S. aureus* for 10 min to 1xMIC of **PYO12** (1 µg/ml) led to strong overexpression of *vraX* and *cwrA* genes (fold-change 629.64 and 736.84, respectively, Fig. 3), genes known to be activated by lipid II binding^18^ or cell-wall targeting antibiotics respectively^20,21^ further suggesting that the mechanism of action for **PYO12** reduces bacterial cell envelope integrity.

Expression data (Fig. 3) of *vraD*,* dltA* and *murZ*,* pbp2* and *sgtB*, suggests that both BraRS and VraRS TCS are also activated by exposure to 1xMIC of **PYO12**^13,23^. As expected, expression of the GraRS-regulated *dltA*^24^ was not significantly changed (fold-change 0.89), most likely due to the neutral charge of **PYO12**. Genes *murZ*,* pbp2* and *sgtB* were moderately upregulated (28.48, 3.28, 2.06-fold change, respectively), indicating VraRS activation due to cell-wall stress. Interestingly, *vraD* was highly upregulated (599-fold), suggesting BraRS is activated. Based on these results, we hypothesized that **PYO12** acts by binding to lipid II or intermediates such as undecaprenyl-pyrophosphate.

Finally, to find further supporting evidence for binding to lipid II, we exposed *B. subtilis* to 1xMIC of **PYO12** (1 µg/ml) for 10 min, and measured expression of *liaI* in comparison to the DMSO control. Exposure to **PYO12** caused a 16-fold induction of *liaI*, which is in the regulon controlled by the LiaRS TCS regulon supporting the lipid II binding theory^25^.

### Binding of PYO12 to undecaprenyl-pyrophosphate

**Fig. 4:**
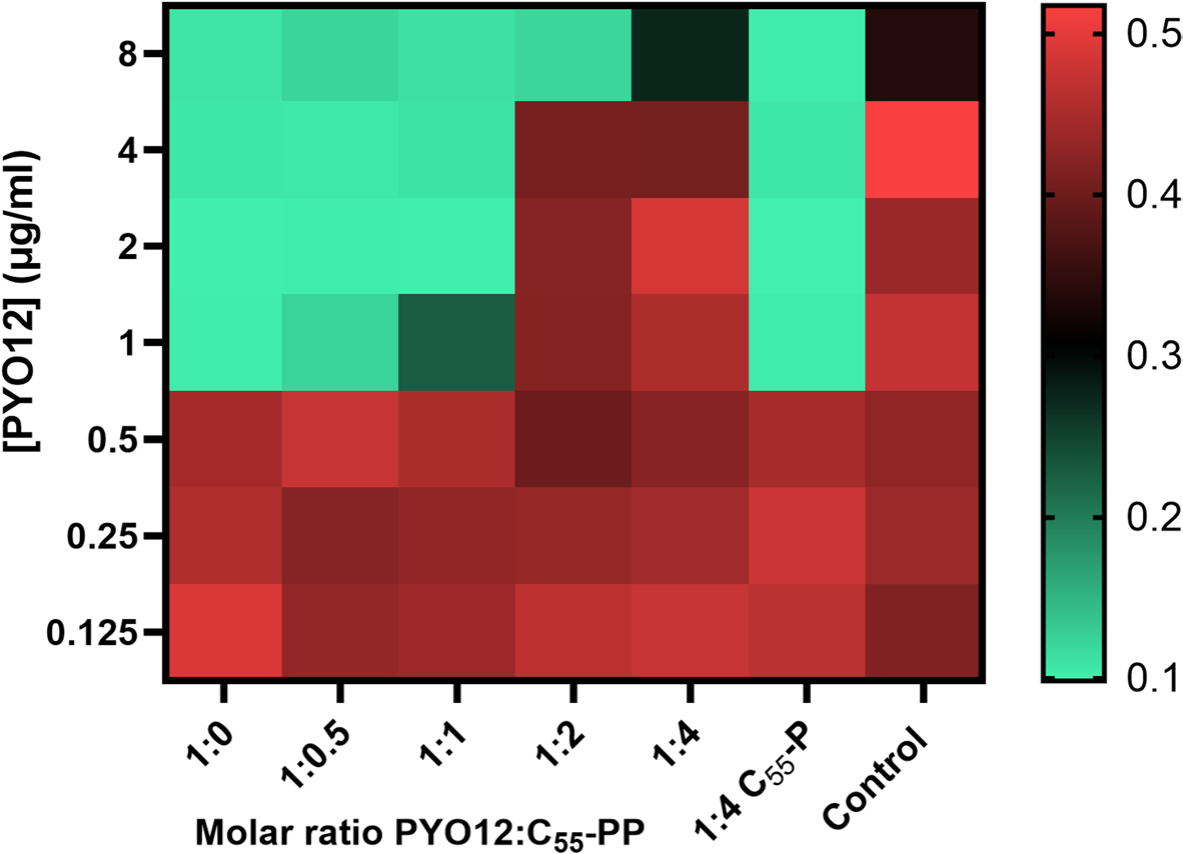
Antagonism of C_55_-PP on antimicrobial activity of **PYO12**. Heat map represents growth of *S. aureus* (OD_600_) in a 96-well plate after exposure to different concentrations of **PYO12** (0.125-8 8 µg/ml) in presence of different molar ratios of C_55_-PP (0 to 4) and C_55_-P (4). Red indicates bacterial growth while green indicates no-growth. Experiments were independently repeated in triplicate and graph shows the average growth per well in these experiments.

To check if **PYO12** binds to undecaprenyl-pyrophosphate(C_55_-PP), we performed a competition assay in which **PYO12** was incubated for 10 min with 0.5-4-fold molar excess of C_55_-PP, before determining its growth inhibitory activity against *S. aureus*. When *S. aureus* is exposed to **PYO12** in the presence of 2-fold molar ratio of the lipid, the MIC increases significantly, suggesting that **PYO12** binds to C_55_-PP and is therefore not available for binding to the bacterial lipid intermediate (Fig. 4). Incubation of **PYO12** with 4-fold molar ratio of the monophosphate C_55−_P no difference in MIC was detected, indicating that the pyrophosphate moiety is necessary for binding. This also indicates that an unspecific binding to lipid is unlikely.

### PYO12 shows low potential for resistance development

No spontaneous resistant mutants were found when inoculating 10^11^ CFU of *S. aureus* Newman onto MHB plates containing 5x and 10x MIC of **PYO12** (5 and 10 µg/ml, respectively), indicating a low potential for spontaneous resistance development. Next, we tested the emergence of resistance mutants when the bacteria were exposed to sub-inhibitory concentrations of **PYO12**. From four experiments, only one resulted in the emergence of a stable resistant mutant with MIC 32 µg/ml after 3 days of exposure to sub-inhibitory concentrations to **PYO12**. Genome sequence analysis revealed the following mutations in the resistant strain: an internal deletion SaeR (1-228 D48-53) and a truncation in YbjH (1-138) (VCF accession number PRJEB86156, EMBL-EBI-EVA), both genes have been related to β-lactam resistance.

## Discussion

Our previous research showed that diphenylpyrazoles (DPP) exert antimicrobial activity by disrupting membrane integrity^19^. However, their toxicity toward mammalian cells, due to disruption of biological membranes and/or targeting of the Hsp90 chaperone, limited optimization. To address this limitation, we modified the DPP scaffold and identified **PYO12**, a phenoxy-phenyl-pyrazol derivative with low haemolytic activity against red blood cells. Further optimization led to **PYO12a** which showed no cytotoxicity against HEK293 cells and only low cytotoxicity against HepG2 cells.

The increased permeability observed in the SYTOX Green assay suggested that **PYO12** causes membrane damage to *S. aureus.* To obtain further evidence that the mechanism of action was related to cell wall or membrane disruption, we quantified expression of genes involved in responses to cell-wall targeting antibiotics. Expression analysis revealed activation of both BraRS and VraRS TCS by **PYO12**. Moderate upregulation of the VraRS regulon suggests that **PYO12** causes cell-wall stress, while strong upregulation of *vraDEH*, similar to that reported for nisin treatment^26^ indicates activation of BraRS, a sensor of lipid II binders. These data suggest that **PYO12** interacts with lipid II or other lipid intermediate^22^. This hypothesis is reinforced by a 16-fold over-expression of *liaI* in *B. subtilis*, a gene specifically induced by the LiaRS membrane stress regulator in presence of lipid II binders^25^. Although the LiaRS regulon is also induced in by organic solvents^25^ the low haemolytic activity of **PYO12** suggests that it does not disrupt biological membranes in a similar fashion. It is noteworthy that previous studies in *S. aureus* treated with novobiocin (bacterial gyrase inhibitor) did not lead to over-expression of any of the aforementioned genes^27^.

Furthermore, competition with 2 M excess of free undecaprenyl pyrophosphate (C_55_-PP) significantly increased the MIC of **PYO12** for *S. aureus*, suggesting that part of **PYO12** binds to C_55_-PP. Binding to lipid C_55_-P was not found, indicating that the pyrophosphate moiety is critical for binding. Similar results were reported for the lipid II targeting small molecules THCz and DCAP^16,17^. These findings suggests that **PYO12** binds to C_55_-PP either directly as a lipid intermediate or as part of lipid II, although direct binding assays are required to confirm lipid II interaction.

**PYO12** displayed potent activity against a panel of Gram-positive pathogens (*E. faecalis vanA*^*+*^, *E. faecium vanA*^+^, *vanB*^*+*^ and *vanAvanB*^*+*^ and MRSA*)*, showing no cross-resistance with lipid II binders vancomycin or methicillin. PYO derivatives had no effect on Gram-negative *E. coli* and *P. aeruginosa.* Improved susceptibility in *E. coli ΔtolC* mutant indicated that efflux pumps play a major role in the lack of activity in Gram-negative bacteria. However, we cannot discard increased sensitivity may be due to other of the pleiotropic effects that can be caused by deletion of TolC. Damage to the inner membrane was also seen in *E. coli* Δ*tolC*, strengthening the hypothesis that **PYO12** specifically disrupts the inner membrane, possibly by binding to lipid II or its intermediates. Time-kill curves showed a dose-dependent bactericidal effect in *S. aureus*, adding to the attractive properties of this compound.

Resistant mutants revealed a deletion in *saeR* that would prevent phosphorylation of this regulator due to the absence of the conserved aspartic acid D51^28^. SaeR regulon is activated in presence of β-lactam antibiotics^29^ and regulate expression of haemolysin and biofilm formation^30^. A truncation was found in *ybjH*, an adaptor protein that promotes proteolysis of Spx regulator^31^. Spx is known to play an important role in β-lactam and vancomycin resistance^32,33^. A non-functional YbjH would lead to an overabundance of Spx and upregulation of genes involved in resistance to cell-wall and membrane damaging antibiotics, consistent with **PYO12** interacting with bacterial membrane. The importance of both genes in the normal virulence and metabolism of *S. aureus* also suggest that in vivo these mutants would be compromised during an infection.

Among the 14 analogues synthesized, only **PYO12a** had improved biological properties, with very low cytotoxicity. The selectivity index of **PYO12a** (S.I. =36) is still below that of clinically relevant antimicrobials such as novobiocin (S.I. = 115, Table 1) so further optimization is necessary. Nevertheless, **PYO12a** shows a substantially improved selectivity index compared to previously reported lipid II targeting small molecules (S.I. 1.2 in DCAP^17^ and 4 THCz^16^. Modifications on the aromatic rings of **PYO12** led to large variations in MIC and cytotoxicity, making the SAR difficult to analyse. As most analogues showed some cytotoxicity, further toxicity studies on different cell types or organoids are needed for **PYO12a** prior to progressing to in vivo experimental models. As these structures are analogues of published DPPs^19^ inhibitors of Hsp90, it is possible that inhibition of Hsp90 is still retained by some of the presented structures. However, the parent molecule **PYO12** did not lead to inhibition of Hsp90 in our assays.

In conclusion, we hypothesize that the small molecule **PYO12** binds to undecaprenyl pyrophosphate containing molecules on the bacterial membrane, leading to membrane disruption and bacterial death. Whether **PYO12** binding to lipid intermediates leads to membrane disruption through pore formation or by intercalation in the membrane is still unknown. The limited activity of **PYO12** against Gram-negative bacteria likely results from efflux from the periplasm before reaching the inner membrane. **PYO12a**, a derivative with similar structure to **PYO12**, exhibited low cytotoxicity for mammalian cells. The discovery of small molecules such as **PYO12a**, which may bind to lipid II or other lipid intermediates while maintaining low mammalian cytotoxic, represents an exciting finding that can open new paths for antibiotic development.

## Materials and methods

### Chemical compounds

Chemicals **PYO1-22** and **PYO12e**, **PYO12f**, **PYO12i** and **PYO12l** were purchased from MolPort (MolPort, Riga, Latvia) in powder form and were > 95% pure by HPLC. Synthesis schemes, purity and characterization of synthetized **PYO23**, **PYO24**, **PYO12**, **PYO12a**, **PYO12b**, **PYO12c**, **PYO12d**, **PYO12g**, **PYO12h**, **PYO12j**, **PYO12k**, **PYO12m**, **PYO12n** can be found in Supplementary Data (Supplementary Figures SF2-SF13). Synthesis was performed by O2H group (Oxford). All compounds were resuspended in dimethyl-sulfoxide (DMSO) to a final concentration of 50 mg/ml. Antibiotics vancomycin, novobiocin and nisin were obtained from Sigma Aldrich.

### Bacterial strains and growth conditions

*Staphylococcus aureus* str. Newman, *S. aureus* USA300 (Methicillin Resistant *S. aureus*, MRSA), *Enterococcus faecalis* (strain E2440), *E. faecalis vanA*^*+*^ (strain E1654), *Enterococcus faecium* (strain E1162), *E. faecium vanA*^*+*^ (strain E0155), *E. faecium vanB*^*+*^ (strain E7422), *E. faecium vanAvanB*^*+*^ (strain E8426), *Bacillus subtilis* str. 168 1A700, *Escherichia coli* ATCC 25,922, *E. coli* D21f2, *E. coli* JW5503^34^ and *Pseudomonas aeruginosa* ATCC 27,853 were grown in Mueller-Hinton Broth (MHB) (Oxoid, Basingstoke, UK) and incubated at 37 ˚C. *Streptococcus suis* P1/7 and *Streptococcus pneumoniae* A66 were cultured in THY media (Todd-Hewitt Broth (Oxoid, Basingstoke, UK) + 0.2% yeast extract (BD Biosciences)) at 37 ˚C and 5% CO_2_.

### Minimal inhibitory concentration (MIC)

The MIC was determined using the microdilution method according to the recommendations of the European committee on antimicrobial susceptibility testing (EUCAST)^35^. Compounds were diluted in 96-well plates (two-fold dilution range 50–0.39 µg/ml) in MHB (THY for *S. suis* and *S. pneumoniae*) in a final volume of 100 µl per well. Bacteria were added as a 1:100 dilution of an overnight culture, 100 µl per well to a final volume of 200 µl per well. Controls were added for bacteria with and without 1% DMSO (vehicle control), and medium alone was added to control for contamination. Plates were incubated for 18 h at 37 ˚C or 37 ˚C and 5% CO_2_ for *S. suis* and *S. pneumoniae.* The lowest concentration where no bacterial growth was detected by optical density at 600 nm (OD_600_) was recorded as MIC.

### Cytotoxicity using alamar blue assay

Human embryonic kidney cells (HEK293, ATCC-CRL-1573) and human hepatocellular carcinoma cells (HepG2, ATCC-HB-8065) were obtained from the American Type Culture Collection (ATCC). Cells were seeded in 96-well plates at a density of 50.000 cells/well in Dulbecco’s Modified Eagle Medium (DMEM) containing glutaMax and phenol red (Gibco) supplemented with 1% penicillin/streptomycin (Gibco) and 10% foetal bovine serum (FBS) (Gibco). Cells were incubated at 37 °C and 5% CO_2_. Exterior wells were filled with PBS to avoid evaporation issues. Once cells reached a confluency of 80–90%, culture media was removed and replaced with 200 µl of exposure media (DMEM without phenol red and antibiotics (Gibco)) containing a 2-fold dilution series of the test compounds (range 50–1.56 µg/ml). FBS was omitted from the culture medium to avoid interaction with the tested compounds. Controls containing only exposure medium, exposure medium with 1% DMSO (vehicle control), exposure medium with 20% DMSO and wells containing no cells were included. After 24-hour exposure at 37 °C and 5% CO_2_, the exposure medium was replaced by 100 µl 10% Alamar Blue (Invitrogen) in exposure medium. Fluorescence (λex = 541 nm, λem = 590 nm) was measured after 45-minutes exposure using Spectramax M5 (Molecular Devices LLC, San Jose, CA, USA). Cell viability compared to the vehicle control was calculated and inhibition curves for each compound were fitted (non-linear curve fit, four variables, bottom constrained = 0) using Prism 9 (Graphpad Software, San Diego, USA). The IC_50_ value for 50% cell viability was calculated based on the fitted curves.

### Haemolysis

Haemolysis protocol was adapted from Evans et al.^36^. Briefly, sheep red blood cells (RBC) 10% washed pool cells in PBS (Rockland Immunochemicals, Limerick, PA, USA) were diluted in PBS to a final concentration of 2% RBCs, and 200 µl were exposed to 500, 250 and 125 µM of compound **PYO12** for 30 min. Non-treated control, 1% DMSO (vehicle control) and complete cell lysis control (Triton-X 2%) were included. Cells were centrifuged 1000 g for 15 min at 4 °C, then 100 µl transferred to a 96-well plate and absorption at 540 nm measured using Spectramax M5. Percentage of haemolysis was calculated:

$$\:Haemolysis=\frac{{A}_{540\:nm,\:test\:condition}-{A}_{540\:nm,\:DMSO\:control}}{{A}_{540\:nm,\:\:TritonX\:Control}-{A}_{540\:nm,\:DMSO\:control}}$$

### Membrane permeability using SYTOX-green

Five hundred microliters of *S. aureus* str. Newman in exponential phase in MHB (OD_600_ = 0.4) were incubated for 5 min with 2, 1 and 0.5 µg/ml of **PYO12**. Nisin was included as a positive control (1x MIC = 6.25 µg/ml) and novobiocin (1xMIC = 0.5 µg/ml), which does not damage the membrane, as a negative control. Non-treated bacteria were also included as negative control. For *E. coli ΔtolC* colistin (1xMIC = 3 µg/ml) was used as positive control. After treatment, cells were centrifuged 4.000 r.p.m. for 5 min, resuspended in filtered PBS and dyed for 5 min in the dark with 0.5 µM of SYTOX^®^ Green (Molecular Probes, Invitrogen). Cells were centrifuged and resuspended with PBS to remove unbound dye. Three hundred microliters of sample were transferred to a 96-well clear-bottom plate (Corning Incorporated). Fluorescence was measured using a CytoFlex^®^ flow cytometer (Beckman Coulter, Brea, CA, USA) using the green (FITC) channel with the excitation wavelength at 488 nm. Overlay histograms were created with CytExpert Software (Bechman Coulter, Brea, CA, USA).

### Time-kill curve

*S. aureus* str. Newman was grown to exponential phase (OD_600_ = 0.25, read in 96-well plate) in MHB media. Five millilitres of exponential culture were treated with 8, 4, 2–1 µg/ml of **PYO12**, a non-treated control was added. At 0, 10, 30 min, 1 h, 2 h,3 h, 4 h and 6 h post-treatment, 20 µl were transferred to 180 µl sterile PBS and 1:10 serial dilutions were made in PBS (dilution factors: 1:10 to 1:10^6^). Five microliters of each dilution were plated in MHB plates. Colonies were counted following overnight incubation at 37 ˚C. Three independent experiments were performed.

### Relative expression using qPCR

*S. aureus* str. Newman or *Bacillus subtilis* str. 168 1A700 were grown to exponential phase (OD_600_ = 0.4) in 50 ml MHB media at 37 °C. Bacteria were divided into four 15 ml Falcon tubes (Corning Incorporated) with 6 ml exponential culture per tube. Two tubes were supplemented with 1% DMSO (vehicle control) and two with 1 µg/ml of **PYO12** for both *S. aureus* and *B. subtilis*. After 10 min at 37 °C cells were centrifuged 4.000 r.p.m. for 10 min at 4 °C, the supernatant was discarded, and the pellet immediately frozen in liquid nitrogen and stored at -80 °C until RNA was extracted.

RNA from the pellet was extracted using RNeasy Mini Kit (QIAGEN) using manufacturer protocol with slight adaptations. Frozen pellet was resuspended in 700 µl of RLT buffer containing 0.1% β-mercaptoethanol. The resuspended pellet was transferred to lysing matrix B 2 ml tubes (MP Biomedicals) and lysis was carried out in a Fast-Prep 24 5G bead beating grinder and lysis system (MP Biomedicals) using the following parameters: All-MetalQuickprep, 2 cycles, 40 s, 6.0 m/sec. Lysing matrix B tubes were centrifuged at 10.000 r.p.m. for 30 s, supernatant transferred to a clean 1.5 ml Eppendorf tube and manufacturer protocol was followed. RNA concentration was determined using Qubit RNA broad range (BR) kit (Invitrogen) in Qubit 4 fluorometer (Invitrogen). cDNA was synthesized from 1 µg RNA using QuantiTect reverse transcription kit (QIAGEN).

Quantitative PCR for relative gene expression was performed in 96-well clear PCR plates (Bio-Rad) and GoTaq qPCR Master Mix (Promega) in a CFX96 Real-Time system (Bio-Rad). Primers (Supplementary Table ST1) were designed using Benchling to amplify 100–200 bp amplicons of the gene of interest. Relative gene expression was calculated using the 2^−ΔΔCt^ method^37^ and data for the *gyrA* gene for normalisation. The experiment, including replicates (*n* = 2) was independently repeated twice.

### Undecaprenyl pyrophosphate antagonist assay

Antagonist assays were performed as reported previously^17^ with small adaptations. Briefly, undecaprenyl-pyrophosphate (C_55_-PP) and monophosphate (Larodan, Solna, Sweden) was mixed with compound **PYO12** in molar ratios of 1:0.5, 1:1, 1:2 and 1:4 (**PYO12**:C_55_-PP) and incubated for 30 min at room-temperature. An MIC test was then performed using the mixed **PYO12**:C_55_-PP as starting compound, with concentrations of **PYO12** ranging from 8 to 0.125 µg/ml. A control including only C_55_-PP was included to ensure that the lipid has no anti-bacterial effect. An extra control was added with C_55_-PP solvent, chloroform: methanol 2:1. Experiments were independently performed in triplicate.

### Resistance development assays

We assessed the emergence of spontaneous resistance by plating 10^11^ CFU of *S. aureus* Newman onto MHB plates containing 5x and 10x MIC concentrations of **PYO12** (5 and 10 µg/ml, respectively) and incubated overnight at 37 °C. Emergence of resistant mutants exposed to sub-inhibitory concentrations of **PYO12** was assessed as follows: tubes containing 2 ml of MHB media supplemented with 0.5, 1 and 2x MIC of **PYO12** were inoculated 1:100 with an overnight culture of *S. aureus* and incubated overnight at 37 °C. The culture that grew at the highest concentration of **PYO12** was then subcultured into tubes containing 0.5, 1, 2x MIC_Previousday_. This was repeated every day until resistance arose. Whole genome sequencing of these resistant cultures was provided by MicrobesNG (https://www.microbesng.com) using Illumina-sequencing. Variant analysis was performed using Galaxy (https://usegalaxy.org/) VarScan tool^38^. VCF files were deposited in the European Variation Archive (EVA) at EMBL-EBI (https://www.ebi.ac.uk/eva/) under accession number PRJEB86156.

## Supplementary Information

Below is the link to the electronic supplementary material.

Supplementary Material 1

## Data Availability

The dataset analysed during the current study are available in the EMBL-EBI-EVA (European Variation Archive) repository, under accession number PRJEB86156.
