# Supplementary material for: Novel antimicrobial 3-phenyl-4-phenoxypyrazole derivatives target cell wall lipid intermediates with low mammalian cytotoxicity
Source: Sci Rep. 2025 Oct 13;15:35646. doi: 10.1038/s41598-025-19561-y (PMC12518513; doi:10.1038/s41598-025-19561-y)
Supplement: Supplementary file 1 — Supplementary Material 1 [file 41598_2025_19561_MOESM1_ESM.pdf]

Novel antimicrobial 3-phenyl-4-phenoxypyrazole derivatives target cell wall lipid intermediates with low mammalian cytotoxicity

Blanca Fernandez-Ciruelos[1,*], Marco Albanese[2,3], Femke Taverne[1], Paul W. Finn[2,3], Jerry M. Wells[1,*]

Supplemental material

Table ST1. Primers used in qPCR.

Primer name	Sequence 5’-3’
qPCR_GyrA_Saureus_Fw	AGCACGTATCGTTGGTGACG
qPCR_GyrA_Saureus_Rv	ACCTTGGCCATCAACAAGCG
qPCR_CwrA_Fw	CATTCTAGGTTTGGTCAAAACG
qPCR_CwrA_Rv	CAGATGGGTTAAATTCTTTCGC
qPCR_VraX_Fw	ATCGTCTTGTAATAAAGAGAGC
qPCR_VraX_Rv	ATTTATCGACAGTATCACCATG
qPCR_VraD_Fw	TGGTTACACATGATCCGGTTGC
qPCR_VraD_Rv	GGCCTGTTTAGAACGTCCTTCC
qPCR_DltA_Fw	TCCCAAGTGCGACGATTTAC
qPCR_DltA_Rv	CTTTCAACGCCAACAGGTAATG
qPCR_SgtB_Fw	CGCCCTCAAGCGTATATTGA
qPCR_SgtB_Rv	TCAACGATTAGCGACAGAGATG
qPCR_Pbp2_Fw	GTAAGAACTTGACTGGTGGATTTG
qPCR_Pbp2_Rv	ACCTGTTACGCCATCAGAATAG
qPCR_MurZ_Fw	TTGCACCTACGCTAACCATATC
qPCR_MurZ_Rv	ATGATGGGTGCCATGTTAGG
qPCR_GyrA_Bsubtilis_Fw	GGAAGAATCACACGTCCTTATCG
qPCR_GyrA_Bsubtilis_Rv	GTACGCCGCTAGCTGTAATAATC
qPCR_LiaI_Fw	TTGGTAGGCAGCAGAAGCC
qPCR_LiaI_Fw	ATCACGGGAATCATCGTGGG

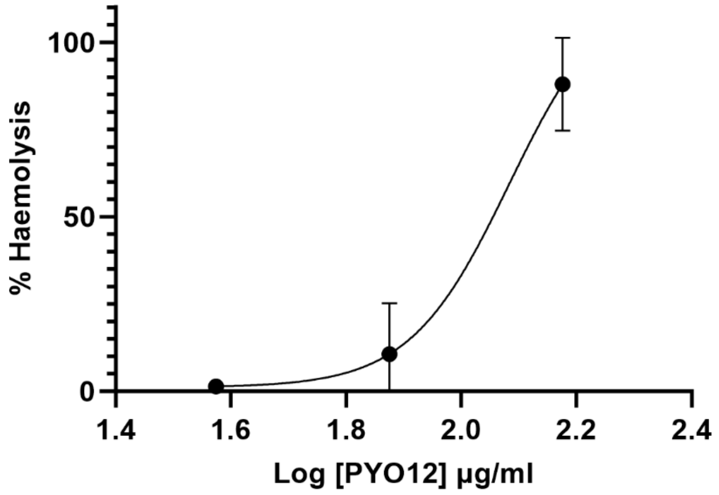

Figure SF1. Haemolysis percentage of PYO12 in RBCs fitted in a non-linear graph used to calculate HC_{50}.

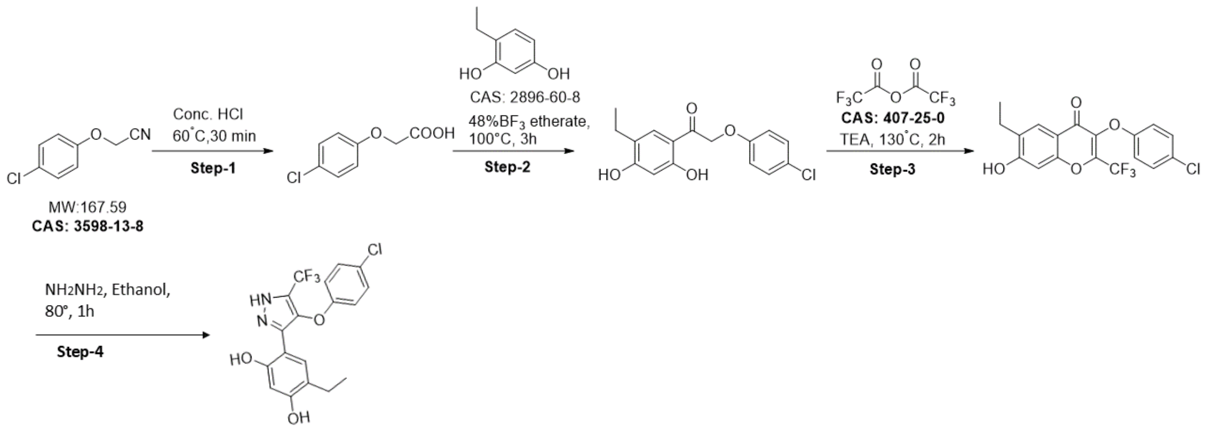

^{1}H NMR (400 MHz, DMSO) δ 13.40 (s, 1H), 10.00 (s, 1H), 9.57 (s, 1H), 7.33 (d, J = 8.8 Hz, 2H), 7.01 (s, 1H), 6.90 (d, J = 8.8 Hz, 2H), 6.44 (s, 1H), 2.29-2.25 (m, 2H), 0.87 (t, J = 7.6 Hz, 3H).

Figure SF2. Synthesis of 4-(4-(4-chlorophenoxy)-5-(trifluoromethyl)-1H-pyrazol-3-yl)-6-ethylbenzene-1,3-diol (PYO23) and ^{1}H NMR results.

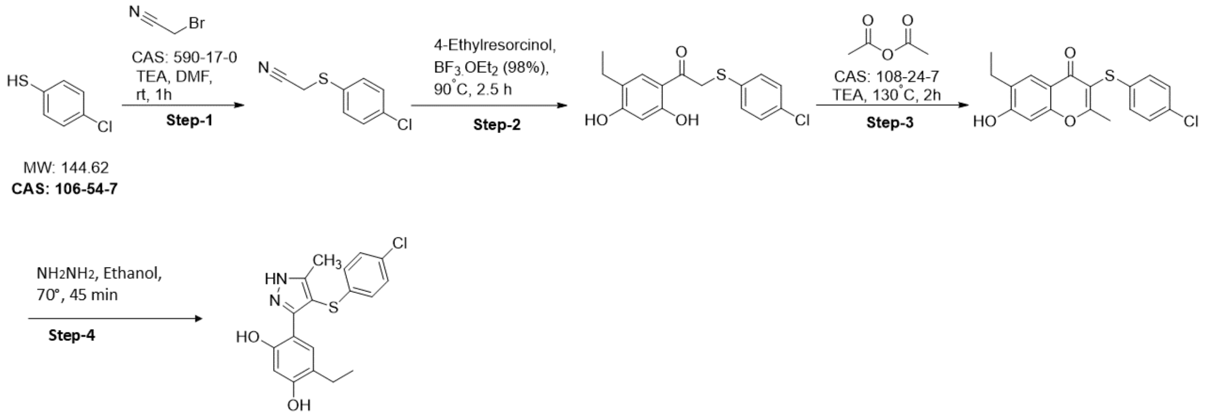

^{1}H NMR (400 MHz, DMSO) δ 13.43 (s, 0.5H), 12.71 (s, 0.5H), 10.82 (s, 0.5H), 9.60-9.42 (m, 1.5H), 7.80 (s, 0.5H), 7.31-7.29 (m, 2H), 7.02-6.95 (m, 2H), 6.43-6.31 (m, 1H), 2.31-2.26 (m, 4H), 2.09 (br s, 1H), 0.82 (br s, 3H).

Figure SF3. Synthesis of 4-(4-((4-chlorophenyl)thio)-5-methyl-1H-pyrazol-3-yl)-6-ethylbenzene-1,3-diol (PYO24) and ^{1}H NMR results.

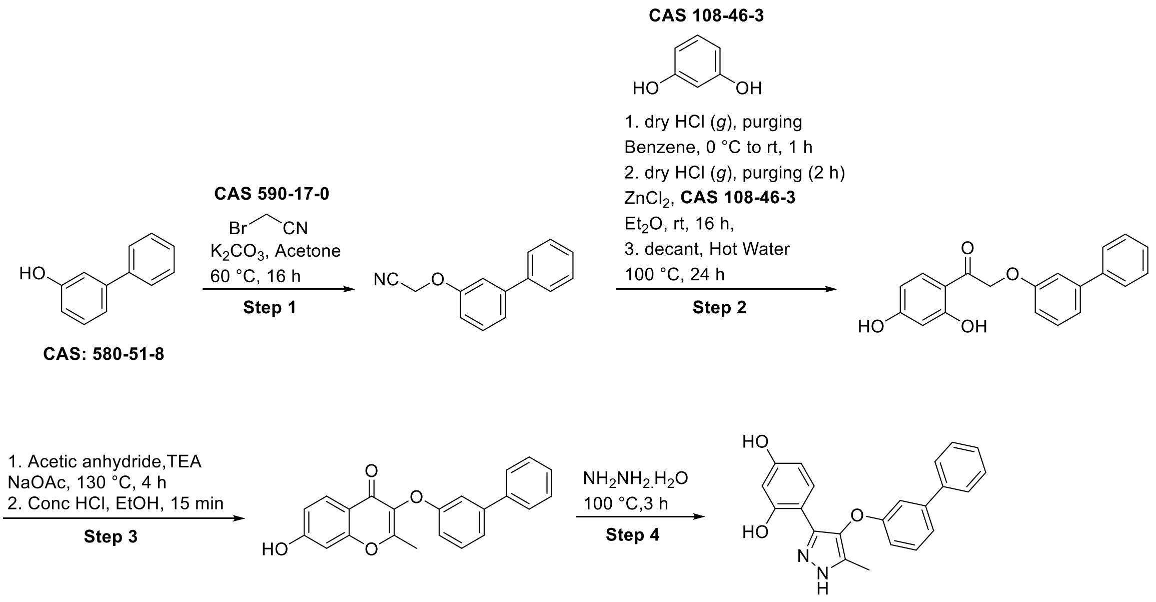

¹H NMR (400 MHz, DMSO-d_6; High temperature): 12.63 (s, 1H), 11.07 (s, 1H), 9.22 (s, 1H), 7.58 (d, J = 7.2 Hz, 2H), 7.46 - 7.29 (m, 6H), 7.19 (s, 1H), 6.90 - 6.88 (m, 1H), 6.32 (bs, 1H), 6.21 - 6.18 (dd, J_1 = 2.4 Hz, J_2 = 8.8 Hz, 1H), 2.12 (bs, 3H)

Figure SF4. Synthesis of 4-(4-([1,1'-biphenyl]-3-yloxy)-5-methyl-1H-pyrazol-3-yl) benzene-1,3-diol (PYO12) and ¹H NMR results.

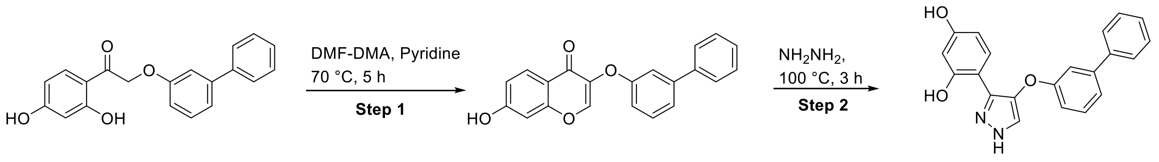

¹H NMR (400 MHz, DMSO-d_6-High temperature): δ 12.55 (bs, 1H), 11.02 (bs, 1H), 9.22 (bs, 1H), 7.78 (bs, 1H), 7.59 (d, J = 7.6 Hz, 2H), 7.50 - 7.31 (m, 6H), 7.27 (s, 1H), 7.00 - 6.98 (m, 1H), 6.35 (bs, 1H), 6.24 (dd, J_1 = 2 Hz, J_2 = 8.4 Hz, 1H)

Figure SF5. Synthesis of 4-(4-([1,1'-biphenyl]-3-yloxy)-1H-pyrazol-3-yl) benzene-1,3-diol (PYO12a) and ¹H NMR results. Synthesis of first intermediate is explained in Figure SF4.

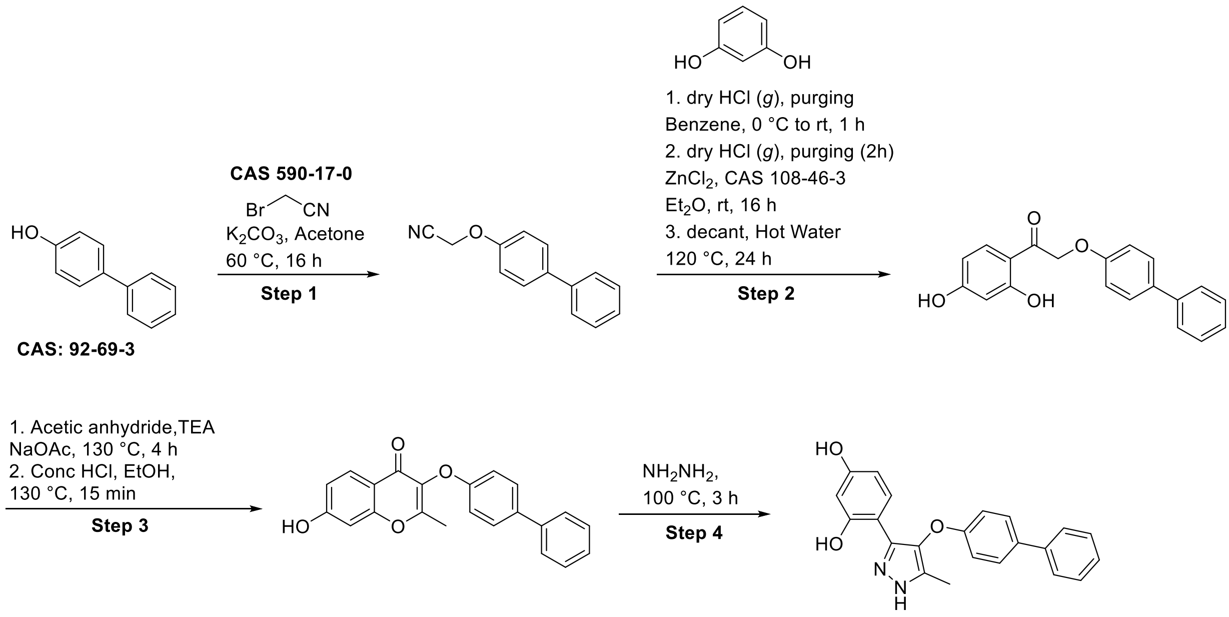

¹H NMR (400 MHz, DMSO-*d*₆- High-temperature): 12.80 (bs, 1H), 10.94 (bs, 1H), 9.21 (s, 1H), 7.60 (d, *J* = 8.4 Hz, 4H), 7.44 - 7.41 (m, 3H), 7.31 (t, *J* = 7.6 Hz, 1H), 7.00 (d, *J* = 8.4 Hz, 2H), 6.32 (s, 1H), 6.18 (dd, J_1 = 2.4 Hz, J_2 = 8.8 Hz, 1H), 2.11 (s, 3H)

Figure SF6. Synthesis of 4-(4-([1,1'-biphenyl]-4-yloxy)-5-methyl-1H-pyrazol-3-yl) benzene-1,3-diol (PYO12g) and ¹H NMR results.

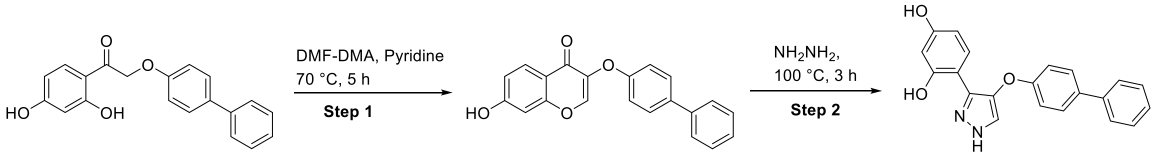

¹H NMR (400 MHz, DMSO-*d*₆; High temperature): *δ* 11.57 (s, 2H), 9.51 (s, 1H), 7.77 (bs, 1H), 7.62 - 7.59 (m, 4H), 7.49 (d, *J* = 8.4 Hz, 1H), 7.43 (t, *J* = 7.6 Hz, 2H), 7.32 (t, *J* = 7.2 Hz, 1H), 7.08 (d, *J* = 8.4 Hz, 2H), 6.36 (bs, 1H), 6.26 - 6.23 (dd, J_1 = 2 Hz, J_2 = 8.4 Hz, 1H)

Figure SF7. Synthesis of 4-(4-([1,1'-biphenyl]-4-yloxy)-1H-pyrazol-3-yl) benzene-1,3-diol (PYO12h) and ¹H NMR results. Synthesis of first intermediate is explained in Figure SF6.

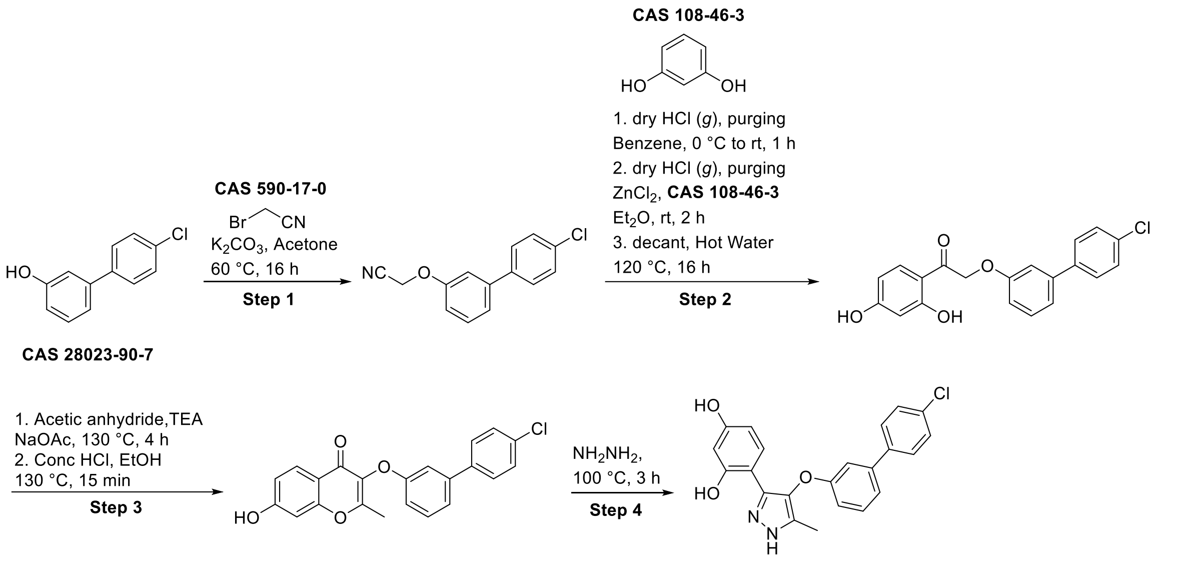

¹H NMR (400 MHz, DMSO-d_6- High-temperature): δ 12.79 (s, 1H), 10.95 (s, 1H), 9.20 (s, 1H), 7.61 (d, J = 8.4 Hz, 2H), 7.48 (d, J = 8.4 Hz, 2H), 7.45 – 7.29 (m, 2H), 7.21 (s, 1H), 6.90 (d, J = 8 Hz, 1H), 6.32 (bs, 1H), 6.19 (d, J = 8.4 Hz, 1H),), 2.12 (bs, 3H)

Figure SF8. Synthesis of 4-(4-((4'-chloro-[1,1'-biphenyl]-3-yl) oxy)-5-methyl-1H-pyrazol-3-yl) benzene-1,3-diol (PYO12b) and ¹H NMR results.

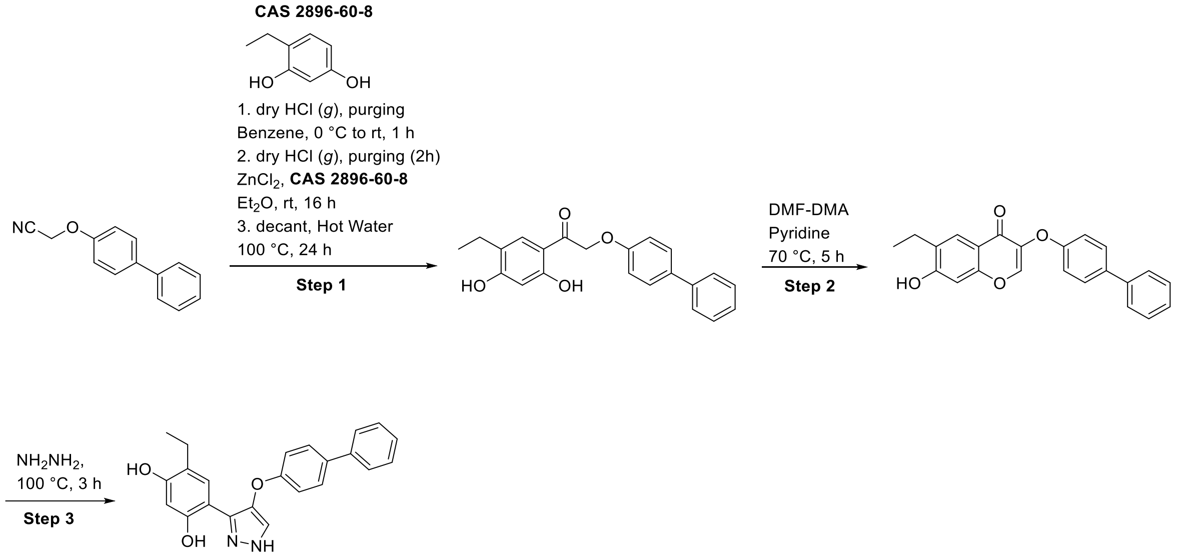

¹H NMR (400 MHz, DMSO-d_6- High-temperature): 12.88 (bs, 1H), 10.64 (bs, 1H), 9.11 (s, 1H), 7.85 (bs, 1H), 7.60 (t, J = 8 Hz, 4H), 7.43 (t, J = 7.6 Hz, 2H) 7.37 - 7.30 (m, 2H), 7.07 (d, J = 8.4 Hz, 2H), 6.39 (s, 1H), 2.36 (q, J = 7.6 Hz, 2H), 0.94 (t, J = 7.2 Hz, 3H)

Figure SF9. Synthesis of 4-(4-([1,1'-biphenyl]-4-yloxy)-1H-pyrazol-3-yl)-6-ethylbenzene-1,3-diol (PYO12j) and ¹H NMR results.

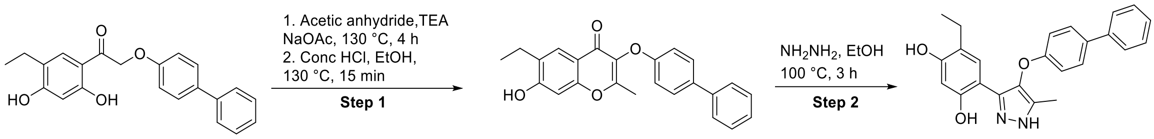

¹H NMR (400 MHz, DMSO-d_6- High temperature): δ 12.73 (s, 1H), 10.60 (s, 1H), 9.05 (s, 1H), 7.61 - 7.57 (m, 4H), 7.42 (t, J = 8 Hz, 2H), 7.32 - 7.29 (m, 2H), 7.0 (d, J = 8.4 Hz, 2H), 6.36 (s, 1H), 2.32 (q, J = 7.2 Hz, 2H), 2.15 (s, 3H), 0.90 (t, J = 7.6 Hz, 3H)

Figure SF10. Synthesis of 4-(4-([1,1'-biphenyl]-4-yloxy)-5-methyl-1H-pyrazol-3-yl)-6-ethylbenzene-1,3-diol (PYO12k) and ¹H NMR results. Synthesis of first intermediate is explained in Figure SF9.

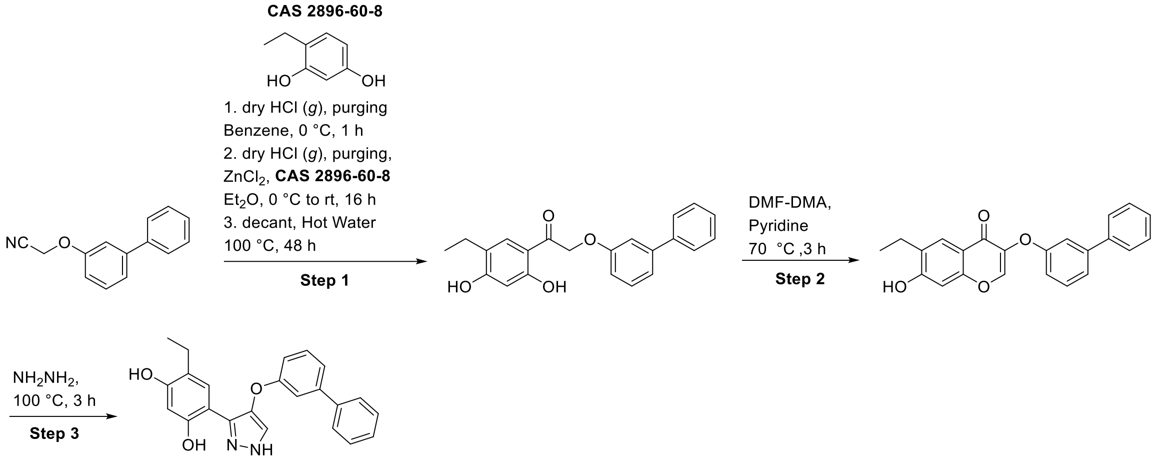

¹H NMR (400 MHz, DMSO-d_6-High-temperature): 12.69 (s, 1H), 10.57 (s, 1H), 9.13 (s, 1H), 7.81 (bs, 1H), 7.57 (d, J = 7.6 Hz, 2H), 7.46 - 7.30 (m, 6H), 7.25 (s, 1H), 6.96 (d, J = 8 Hz, 1H), 6.38 (s, 1H), 2.37 - 2.31 (m, 2H), 0.91 (t, J = 7.2 Hz, 3H)

Figure SF11. Synthesis of 4-(4-([1,1'-biphenyl]-3-yloxy)-1H-pyrazol-3-yl)-6-ethylbenzene-1,3-diol (PYO12c) and ¹H NMR results

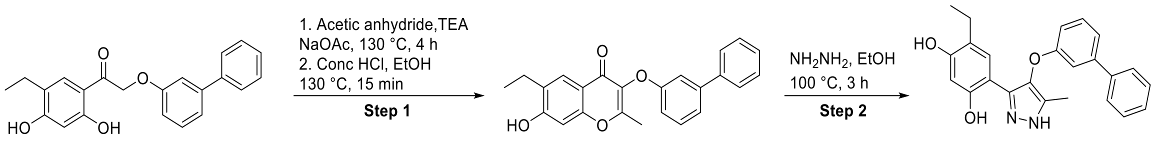

¹H NMR (400 MHz, DMSO-d_6- High-Temperature): δ 12.76 (s, 1H), 10.70 (s, 1H), 9.05 (s, 1H), 7.56 (d, J = 8 Hz, 2H), 7.44 (t, J = 7.6 Hz, 2H), 7.40 - 7.28 (m, 4H), 7.20 (s, 1H), 6.88 (d, J = 8 Hz, 1H), 6.33 (s, 1H), 2.33 - 2.28 (m, 2H), 2.17 (s, 3H), 0.86 (t, J = 8.8 Hz, 3H).

Figure SF12. Synthesis of 4-(4-([1,1'-biphenyl]-3-yloxy)-5-methyl-1H-pyrazol-3-yl)-6-ethylbenzene-1,3-diol (PYO12d) and ¹H NMR results. Synthesis of first intermediate is explained in Figure SF11.

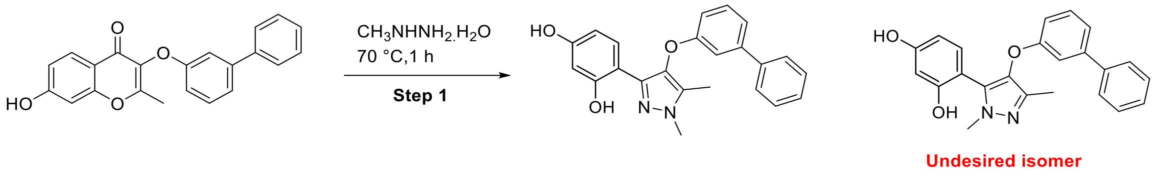

¹H NMR (400 MHz, DMSO-d_6): δ 10.81 (s, 1H), 9.50 (s, 1H), 7.60 (d, J = 7.6 Hz, 2H), 7.47- 7.32 (m, 6H), 7.24 (s, 1H), 6.88 (d, J = 8 Hz, 1H), 6.28 (s, 1H), 6.20 - 6.17 (m, 1H), 3.83 (s, 3H), 2.14 (s, 3H)

Figure SF13. Synthesis of of 4-(4-([1,1'-biphenyl]-3-yloxy)-1,5-dimethyl-1H-pyrazol-3-yl)benzene-1,3-diol (PYO12m and PYO12n) and the undesired isomer 4-(4-([1,1'-biphenyl]-3-yloxy)-2,5-dimethyl-1H-pyrazol-3-yl)benzene-1,3-diol (PYO12n) ¹H NMR results. Synthesis of first intermediate is explained in Figure SF11.
